# Significant receptor affinities of metabolites and a degradation product of mometasone furoate

**DOI:** 10.1186/1465-9921-5-7

**Published:** 2004-07-22

**Authors:** Anagnostis Valotis, Petra Högger

**Affiliations:** 1 Institut für Pharmazie und Lebensmittelchemie, Bayerische Julius-Maximilians-Universität, Würzburg, Germany

## Abstract

Mometasone furoate (MF) is a highly potent glucocorticoid used topically to treat inflammation in the lung, nose and on the skin. However, so far no information has been published on the human glucocorticoid receptor activity of the metabolites or degradation products of MF. We have now determined the relative receptor binding affinities of the known metabolite 6β-OH MF and the degradation product 9,11-epoxy MF to understand their possible contribution to undesirable systemic side effects. In competition experiments with human lung glucocorticoid receptors we have determined the relative receptor affinities (RRA) of these substances with reference to dexamethasone (RRA = 100). We have discovered that 6β-OH MF and 9,11-epoxy MF display RRAs of 206 ± 15 and 220 ± 22, respectively. This level of activity is similar to that of the clinically used inhaled corticosteroid flunisolide (RRA 180 ± 11). Furthermore we observed that 9,11-epoxy MF is a chemically reactive metabolite. In recovery experiments with human plasma and lung tissue we found a time dependent decrease in extractability of the compound. Hence, we provide data that might contribute to the understanding of the pharmacokinetics as well as the clinical effects of MF.

## Introduction

Mometasone furoate (MF) is a highly potent topical glucocorticoid for the treatment of asthma [1], allergic rhinitis [2] and various skin diseases [3]. The clinical efficacy of MF is comparable to that of fluticasone propionate [4]. Both compounds have a very high affinity to the human glucocorticoid receptor. With reference to dexamethasone, fluticasone propionate has an eighteen-fold higher relative receptor affinity (RRA) of 1800 [5,6], while MF displays a RRA of about 2200 [7]. These high receptor affinities as well as the administered doses, the absolute lung deposition and a prolonged retention time in the lung tissue contribute to the clinical success of both compounds.

Besides the efficacy of a corticosteroid, safety issues have to be taken into consideration. For topically applied glucocorticoids, the high local anti-inflammatory activity should be paralleled by a low systemic exposure. Therefore, a prolonged redistribution from lung tissue into systemic circulation and a rapid and complete hepatic metabolism of the compounds to inactive derivatives are favorable. For MF, a very low systemic bioavailability of less than 1 % has been reported [8]. However, there have been discussions about the appropriate methodology and the validity of the conclusion has been questioned [9,10]. Indeed, the claimed low systemic bioavailability of MF would appear to be inconsistent with the considerable suppression of the hypothalamic-pituitary-adrenal (HPA) axis recorded in a clinical study [11,12]. Frequently, various researchers called attention to the formation of active MF metabolites that would account for undesirable systemic side effects [9,13]. In an early study by Isogai et al. more than ten different metabolites and related compounds of MF displayed varying binding affinities to the rat glucocorticoid receptor [14].

There had been, however, not much information on the extent and site of metabolite formation in humans. Recent studies now provided some of the required information [7,13,15,16]. In rat liver microsomes, 6β-hydroxy MF (6β-OH MF) was identified as the major metabolite [16]. This metabolite was also found after incubation of MF with human liver and intestine microsomes [13]. Additionally, the degradation product 9,11-epoxy MF was detected in plasma and urine. 9,11-epoxy MF is formed in aqueous solutions [15] indicating a general time- and pH-dependent instability of MF [7]. Recently, we discovered 9,11-epoxy MF in incubation mixtures of human lung tissue as well as in fresh human plasma [7]. We pointed out that this degradation product might form covalent adducts with proteins in follow-up reactions.

Despite the recent discovery of the major metabolite 6β-OH MF and the abundant degradation product 9,11-epoxy MF it is still not clear whether these compounds retain any significant binding affinity to the human glucocorticoid receptor. In the present study we addressed this open question and we present some evidence that the degradation product might bind tightly, most possibly covalently, to protein structures in human lung tissue and plasma.

## Materials and Methods

### Chemicals and reagents

Mometasone furoate (MF), 6-hydroxy mometasone furoate (6-OH MF), mometasone and 9,11-epoxy mometasone furoate (9,11-epoxy MF) were generous gifts from GlaxoSmithKline (Greenford, England). [^3^H]-Dexamethasone was obtained from Amersham (Freiburg, Germany). All other chemicals were obtained from Sigma-Aldrich-Chemie (Taufkirchen, Germany) or E. Merck (Darmstadt, Germany).

### Source and handling of human specimen

Human lung tissue resection material was obtained from patients with bronchial carcinomas who gave informed consent. Cancer-free tissue was used for the experiments. None of the patients was treated with glucocorticoids for the last 4 weeks prior to surgery. Tissue samples were shock frozen in liquid nitrogen after resection and stored at -70°C until usage. To collect sufficient material for the experiments, tissue samples of three or more patients were pooled. Lung cytosol for receptor competition experiments was prepared as detailed in [6]. Plasma samples were obtained from healthy volunteers who gave informed consent. Samples were either used immediately or were shock frozen in liquid nitrogen and stored at -70°C until usage.

### Determination of relative receptor affinity by competition tests

The competition experiments were performed according to the procedure described earlier [6]. The displacement of a constant concentration of [^3^H] labelled dexamethasone by various concentrations of 6-OH MF, mometasone and 9,11-epoxy MF was determined.

### Recovery of MF and 9,11-epoxy MF from human plasma, lung tissue and buffer

MF or 9,11-epoxy MF, respectively, were added to human plasma, lung tissue suspension (0.5 g / 20 ml) or buffer (0.2 M phosphate buffer, pH 7.4) yielding an initial concentration of 0.3 μg/ml. Only glass lab ware was used for these experiments to exclude any non-specific binding effects of the highly lipophilic compounds to plastic material. Samples were incubated at 37°C in a shaking water bath. At designated time intervals samples of 1.0 ml were removed, subjected to a fluid extraction with diethylether and analyzed by HPLC.

### Sample preparation and HPLC conditions

Samples were prepared and analyzed as described previously [7]. The HPLC system consisted of a Waters HPLC (Milford, MA) with a 1525 binary pump, a 717plus autosampler and 2487 dual wavelength absorbance detector set at the detection wavelength of 254 nm. Data collection and integration were accomplished using Breeze™ software version 3.2. Analysis was performed on a Symmetry C_18 _column (150 × 4.6 mm I.D., 5 μm particle size, Waters, MA).

## Results

We determined the relative receptor affinities (RRAs) of 6β-OH MF, 9,11-epoxy MF and mometasone base by competition assays with reference to dexamethasone (RRA = 100). Both, the metabolite 6β-OH MF and the degradation product 9,11-epoxy MF displayed residual receptor binding affinities about twice as high as dexamethasone (Table 1). This level of activity is between that of the clinically used inhaled corticosteroids flunisolide (RRA 180 ± 11) and triamcinolone acetonide (RRA 361 ± 26) [5]. Mometasone which is formed by hydrolysis of the furoate ester, revealed an even higher RRA of almost 800. For comparison, the RRA of the parent compound MF is about 2200 [7].

**Table 1:** Relative receptor affinities of mometasone furoate (MF, data from [7]), its metabolites 6β-hydroxy mometasone furoate (6β-OH MF), mometasone and the major degradation product 9,11-epoxy mometasone furoate (9,11-epoxy MF) in relation to dexamethasone (Dexa). Values represent mean and mean deviation of the mean of n = 3 independent experiments.

Compound	Relative receptor affinity (RRA)	Mean deviation of the mean
MF	2244	± 142
Dexa	100	± 10
6β-OH MF	206	± 15
9,11-epoxy MF	220	± 22
Mometasone	781	± 27

**Figure 1:**
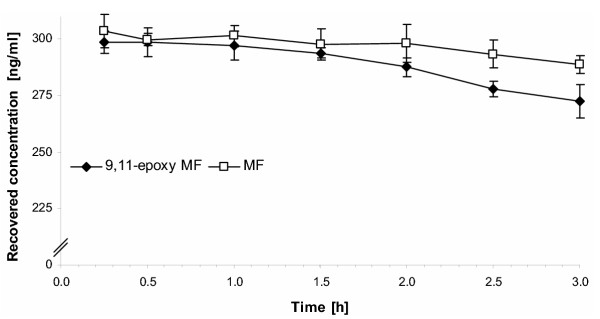
Recovery of mometasone furoate (MF) and its degradation product 9,11-epoxy MF from incubation mixtures with human plasma over three hours. Each data point represents the mean and mean deviation of the mean of three experiments.

To investigate the putative reactivity of the degradation product 9,11-epoxy MF we monitored the recovery of MF and 9,11-epoxy MF from human plasma by organic solvent extraction (Fig. 1). The determination of recovery was limited to a period of three hours since MF is successively degraded to 9,11-epoxy MF [7]. The retrieval of 9,11-epoxy MF from human plasma decreased steadily and was clearly more pronounced than for MF. After three hours 9.14 ± 2.3 % of 9,11-epoxy MF was not recovered from plasma while 4.8 ± 1.4 % of MF was not extractable any more.

**Figure 2:**
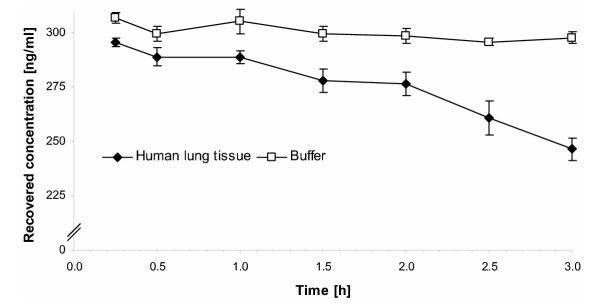
Recovery of 9,11-epoxy MF from incubation mixtures with human lung tissue and buffer (control experiment) over three hours. Each data point represents the mean and mean deviation of the mean of three experiments.

The decrease in recovery of 9,11-epoxy MF from human lung tissue was even more evident (Fig. 2). While there was no change in the control incubation mixture comprising of buffer (pH 7.4) a pronounced and steady decrease in recovery rates of 9,11-epoxy MF was revealed. After three hours 16.61 ± 0.58 % of the degradation product was not extractable any more. No new peaks were observed in the HPLC to indicate a further degradation of 9,11-epoxy MF.

## Discussion

In the present study we have determined the relative receptor binding affinities of the mometasone furoate (MF) metabolite 6β-OH MF and its degradation product 9,11-epoxy MF to understand their possible contribution to undesirable systemic side effects. For the first time we provide data that both compounds are significantly active at the human glucocorticoid receptor with binding affinities twice as high as dexamethasone and similar to that of the clinically used inhaled corticosteroids flunisolide and triamcinolone acetonide [5]. Furthermore, our data demonstrate that the ubiquitous degradation product 9,11-epoxy MF undergoes follow-up reactions.

Glucocorticoids currently used for topical application in asthma therapy all share the safety relevant property of extensive metabolism and formation of inactive metabolites. For MF, however, data was sparse so far. Though putative metabolites and degradation products with binding affinity to the rat glucocorticoid receptor have been previously suggested [14], it was not clear whether this might have any implications to humans. Potential human metabolites such as 6β-OH MF, mometasone or MF-epoxide have been proposed [8], but experimental evidence of *in vivo *formation of these compounds was still lacking. Studies of Teng *et al. *identified 6β-OH MF and 9,11-epoxy MF as candidate compounds that can indeed be formed *in vivo *either by hepatic metabolism or by simple degradation of MF [13,16]. We discovered that 9,11-epoxy MF is also formed in human lung tissue suspensions and plasma [7].

Usually hydroxylation at the 6β position results in inactivation of the corticosteroid. The 6-OH metabolite of various glucocorticoids displays little or no residual binding affinity to the receptor (e.g.) [17,18]. This, however, is different for MF with its 6β-OH metabolite exhibiting a relative receptor affinity of more than 200 (dexamethasone: 100). Obviously, the substitution pattern of the D-ring of MF confers such potent binding affinity that hydroxylation in 6β position does not result in complete inactivation of this corticosteroid. Notably, neither the RRA we determined for 6β-OH MF nor for mometasone are coherent with the binding results of the early studies with the rat glucocorticoid receptors [14]. This emphasizes the need for data derived from human receptor studies.

The MF degradation product 9,11-epoxy MF also displays a significant receptor binding affinity with an RRA of about 200. This RRA is within the range that could be expected from the studies of Isogai *et al*. [14]. Since 9,11-epoxy MF is also formed in the lung tissue suspensions [7], it can be assumed that it contributes to the effects after inhalation of MF. It can, however, be predicted that this compound might be also responsible for undesired effects such as HPA axis suppression.

Besides the significant residual receptor binding affinity of 9,11-epoxy MF we discovered that this compound undergoes follow-up reactions. After incubation with plasma clearly less of 9,11-epoxy MF compared to the parent compound MF was recovered by extraction with an organic solvent. This extraction procedure usually reliably retrieves all non-covalently bound substance from the incubation mixture. In human lung tissue, it was even more obvious that 9,11-epoxy MF was recovered completely from buffer, but not from the tissue suspension. About 17 % of 9,11-epoxy MF was "lost" after three hours of incubation. This observation cannot be explained by simple non-specific tissue binding since the tissue adsorption reaches equilibrium very quickly after about 20 min [7]. Also, the non-specifically bound compound would be still extractable by organic solvents. Generally, epoxides are chemically reactive molecules that tend to bind irreversibly to cellular macromolecules. If this were the case for 9,11-epoxy MF it would have two implications. Firstly, irreversibly bound 9,11-epoxy MF escapes detection and feigns a low bioavailability after inhalation. The fact that after inhalation of a single dose of tritium labelled MF only 88% (63–99 %) of total radioactivity was recovered over seven days in humans [8] seems to support this conclusion. Secondly, if 9,11-epoxy MF is indeed covalently bound to cellular macromolecules the adduct might lead to allergic reactions. Such reactions to corticosteroids for asthma therapy do occur occasionally [19]. However, it cannot be excluded that 9,11-epoxy MF is further degraded although we did not observe any new peaks that emerged in the HPLC chromatograms. The chromatographic conditions were chosen for rather lipophilic compounds, thus, if a further degradation product of 9,11-epoxy MF with pronounced hydrophilic character was formed, it might have escaped our attention. However, the possibility of covalent adduct formation of 9,11-epoxy MF should be further investigated.

## Conclusions

In contrast to other inhaled corticosteroids MF generates an active metabolite, 6β-OH MF, in the liver. The degradation product 9,11-epoxy MF, which is formed in human lung tissue and plasma, exhibits significant receptor affinity as well. Additionally, we found that 9,11-epoxy MF undergoes follow-up reactions. Our data contribute to the understanding of how the claimed low bioavailability of MF parent compound after inhalation might still be accompanied by HPA axis suppression. Thus, our findings are consistent with both pharmacokinetic and clinical data. We strongly suggest a clinical trial that determines both efficacy and safety in parallel as well as all known metabolites and degradation products after application of MF.

## Authors' contributions

AV carried out all experiments and the data analysis and participated in the design of the study. PH conceived of and designed the study and wrote the manuscript. All authors read and approved the final manuscript.

## References

[B1] Sharpe M, Jarvis B (2001). Inhaled mometasone furoate: a review of its use in adults and adolescents with persistent asthma. Drugs.

[B2] Trangsrud AJ, Whitaker AL, Small RE (2002). Intranasal corticosteroids for allergic rhinitis. Pharmacotherapy.

[B3] Brazzini B, Pimpinelli N (2002). New and established topical corticosteroids in dermatology: clinical pharmacology and therapeutic use. Am J Clin Dermatol.

[B4] O'Connor B, Bonnaud G, Haahtela T, Luna JM, Querfurt H, Wegener T, Lutsky BN (2001). Dose-ranging study of mometasone furoate dry powder inhaler in the treatment of moderate persistent asthma using fluticasone propionate as an active comparator. Ann Allergy Asthma Immunol.

[B5] Würthwein G, Rehder S, Rohdewald P (1992). Lipophilicity and receptor affinity of glucocorticoids. Pharm Ztg Wiss.

[B6] Hogger P, Rohdewald P (1994). Binding kinetics of fluticasone propionate to the human glucocorticoid receptor. Steroids.

[B7] Valotis A, Neukam K, Ehlert O, Högger P (2004). Human receptor kinetics, tissue binding affinity and stability of mometasone fuorate. J Pharm Sci.

[B8] Affrime MB, Cuss F, Padhi D, Wirth M, Pai S, Clement RP, Lim J, Kantesaria B, Alton K, Cayen MN (2000). Bioavailability and metabolism of mometasone furoate following administration by metered-dose and dry-powder inhalers in healthy human volunteers. J Clin Pharmacol.

[B9] Derendorf H, Daley-Yates PT, Pierre LN, Efthimiou J (2001). Systemic bioavailability of inhaled steroids: the importance of appropriate and comparable methodology. Eur Respir J.

[B10] Derendorf H, Daley-Yates PT, Pierre LN, Efthimiou J (2002). Bioavailability and metabolism of mometasone furoate: pharmacology versus methodology. J Clin Pharmacol.

[B11] Affrime MB, Kosoglou T, Thonoor CM, Flannery BE, Herron JM (2000). Mometasone furoate has minimal effects on the hypothalamic-pituitary-adrenal axis when delivered at high doses. Chest.

[B12] Lipworth BJ (2001). Mometasone furoate levels. Chest.

[B13] Teng XW, Cutler DJ, Davies NM (2003). Mometasone furoate degradation and metabolism in human biological fluids and tissues. Biopharm Drug Dispos.

[B14] Isogai M, Shimizu H, Esumi Y, Terasawa T, Okada T, Sugeno K (1993). Binding affinities of mometasone furoate and related compounds including its metabolites for the glucocorticoid receptor of rat skin tissue. J Steroid Biochem Mol Biol.

[B15] Teng XW, Cutler DC, Davies NM (2003). Degradation kinetics of mometasone furoate in aqueous systems. Int J Pharm.

[B16] Teng XW, Cutler DJ, Davies NM, Cutler DC (2003). Kinetics of metabolism and degradation of mometasone furoate in rat biological fluids and tissues. J Pharm Pharmacol.

[B17] Hochhaus G, Moellmann HW (1990). Binding affinities of rimexolone (ORG 6216), flunisolide and their putative metabolites for the glucocorticoid receptor of human synovial tissue. Agents Actions.

[B18] Grogan WM, Phillips VM, Schuetz EG, Guzelian PS, Watlington CO (1990). Corticosterone 6 beta-hydroxylase in A6 epithelia: a steroid-inducible cytochrome P-450. Am J Physiol.

[B19] Kilpio K, Hannuksela M (2003). Corticosteroid allergy in asthma. Allergy.

